# Design of Imide Oligomer‐Mediated MOF Clusters for Solar Cell Encapsulation Systems by Interface Fusion Strategy

**DOI:** 10.1002/advs.202206748

**Published:** 2023-01-29

**Authors:** Minghong Wu, Jiaxin Li, Xi Zhang, Shumei Liu, Jianqing Zhao, Weiqi Xie

**Affiliations:** ^1^ School of Materials Science and Engineering State Key Laboratory of Luminescent Materials and Devices South China University of Technology Guangzhou 510640 P. R. China; ^2^ School of Minerals Processing and Bioengineering Central South University Changsha 410083 P. R. China

**Keywords:** interface fusion, metal‐organic framework (MOF) clusters, solar cells, tunable haze

## Abstract

Dielectric encapsulation materials are promising for solar cell areas, but the unsatisfactory light‐management capability and relatively poor dielectric properties restrict their further applications in photovoltaic and microelectronic devices. Herein, an interface fusion strategy to engineer the interface of MOF (UiO‐66‐NH_2_) with anhydride terminated imide oligomer (6FDA‐TFMB) is designed and a novel MOF cluster (UFT) with enhanced forward scattering and robust porosity is prepared. UFT is applied as an optical and dielectric modifier for bisphenol A epoxy resin (DGEBA), and UFT epoxy composites with high transmittance (>80%), tunable haze (45–58%) and excellent dielectric properties can be prepared at low UFT contents (0.5–1 wt%), which delivers an optimal design for dielectric encapsulation systems with efficient light management in solar cells. Additionally, UFT epoxy composites also show excellent UV blocking, and hydrophobic, thermal and mechanical properties. This work provides a template for the synthesis of covalent bond‐mediated nanofillers and for the modulation of haze and dielectric properties of dielectric encapsulation materials for energy systems, semiconductors, microelectronics, and more.

## Graphical abstract

A novel core–shell nanofiller UFT with enhanced forward scattering and robust porosity is achieved by using an interfacial fusion strategy to engineer the interface of MOFs with imide oligomers, thus creatively endowing the UFT encapsulation materials with outstanding light‐management function (tunable haze and forward scattering) and simultaneously enhanced dielectric properties, which show promising applications in solar cells.

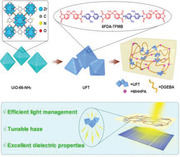

## 1 Introduction

Epoxy thermosets are one kind of the commonly used encapsulation materials for solar cells due to their high transparency, heat resistance, and excellent mechanical properties.^[^ ^1^ ^]^ However, the extremely low haze (<2%) makes them difficulty to achieve efficient light management. Previous studies^[^ ^2^ ^]^ revealed that optical materials with high transmittance and high haze can improve light absorption by increasing the path of scattered light through the active solar layers, thereby enhancing the short‐circuit current (*I*_sc_) and power conversion efficiency (PCE). Nevertheless, the realization of epoxy thermosets with high transmittance and high haze as the light managers of solar cells is still in its infancy. In addition, low dielectric and insulating properties of encapsulation materials also play an important role in the field of photovoltaic devices, semiconductors, and microelectronics.^[^ ^3^ ^]^ Therefore, it is of great significance and remains an ongoing challenge to develop dielectric epoxy encapsulation materials combining high light transmission and high haze.

Various nanofillers (i.e., SiO_2_, alumina nanowire, and nanocellulose) have been utilized to improve the haze and scattering ability of matrices.^[^ ^4^ ^]^ Among them, metal‐organic frameworks (MOFs) were employed as scattering media for the preparation of optical materials with high transmittance and high haze,^[^ ^5^ ^]^ indicating their potential application in the light managers of solar cells. Besides, MOFs are also promoted as next‐generation low dielectric materials due to the porosity and insulation.^[^ ^6^ ^]^ Hence, MOFs show great potential for the preparation of dielectric light diffusers. As one of the most stable and versatile MOFs,^[^ ^7^ ^]^ UiO‐66‐NH_2_ is widely applied in gas adsorption,^[^ ^8^ ^]^ catalysis^[^ ^9^ ^]^ and drug delivery.^[^ ^10^ ^]^ However, to the best of our knowledge, no studies have been conducted on applying UiO‐66‐NH_2_ to the light management of solar devices. Nevertheless, the exposed pore structure of UiO‐66‐NH_2_ is also susceptible to the penetration of small molecules (i.e., curing agent MHHPA, epoxy prepolymer, and water),^[^ ^11^ ^]^ which destroys the porosity and changes the optical properties (e.g., refractive index), thereby impairing dielectric property and scattering ability. Furthermore, previous studies^[^ ^12^ ^]^ usually introduced MOFs directly into polymer matrices to prepare low dielectric materials, which may lead to the poor interfacial compatibility and large interfacial polarization. Therefore, reliable strategies are required to achieve efficient light management and maintain the excellent dielectric property of UiO‐66‐NH_2_.

**Figure 1:**
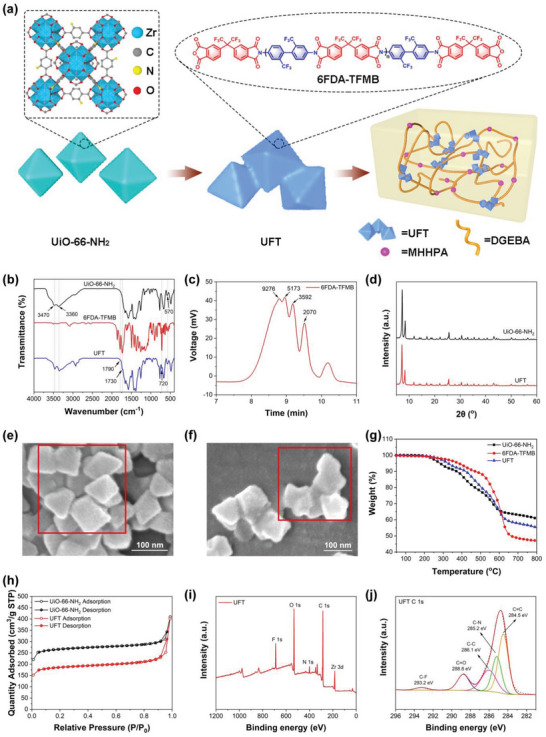
a) Schematic diagram of the preparation process of UFT composites. b) FT‐IR spectra of UiO‐66‐NH_2_, 6FDA‐TFMB, and UFT. c) GPC curve of 6FDA‐TFMB. d) XRD spectra of UiO‐66‐NH_2_ and UFT. e) SEM micrograph of UiO‐66‐NH_2_. f) SEM micrograph of UFT. g) TGA plots of UiO‐66‐NH_2_, 6FDA‐TFMB, and UFT under N_2_ atmosphere. h) N_2_ adsorption–desorption isotherms of UiO‐66‐NH_2_ and UFT. i) XPS spectra of UFT and j) its high‐resolution C1s spectra.

In this work, we first introduce a new strategy that utilizes an anhydride‐terminated imide oligomer 6FDA‐TFMB to engineer the MOF surface and achieve the interface fusion. A novel MOF cluster (UiO‐66‐NH_2_@6FDA‐TFMB, UFT) is self‐assembled by 6FDA‐TFMB and several particles of UiO‐66‐NH_2_. With the aid of the interface fusion strategy, 6FDA‐TFMB acts as a linker to enlarge the particles of UFT to several hundred nanometers, which leads to the significant forward scattering according to Mie scattering theory.^[^ ^13^ ^]^ It is worth noting that the unique core–shell structure of UFT also protects UiO‐66‐HN_2_ from penetration by small molecules, ensuring the high porosity of UiO‐66‐HN_2_ and maintaining the disparity in the optical properties between the matrix and UFT. Furthermore, the anhydride groups on the UFT surface also promote the compatibility between UFT and epoxy matrix, which is essential for the formation of uniform haze in optical materials and the reduction of interfacial polarization in dielectric materials. Therefore, UFT is applied as an optical and low dielectric modifier for bisphenol A epoxy resin DGEBA (**Figure** **1**a), and UFT epoxy composites with high transmittance (>80%), tunable haze (45–58%) and excellent dielectric properties are prepared at low UFT contents (0.5–1 wt%), which also enhance the PCE of silicon‐based solar cells from 14.67% to 15.64% through efficient light management. Moreover, UFT epoxy composites also exhibit excellent UV blocking, hydrophobic, thermal, and mechanical properties. This design principle, in which nanofillers are interfacially fused to form unique cluster structures with enhanced forward scattering and robust porosity, can further inspire the self‐assembly of covalent bond‐mediated nanostructures and expand the applications in the fields that require the efficient optical management and excellent dielectric properties such as energy systems, semiconductors and microelectronics.

## 2 Results and Discussion

### 2.1 Characterization of UFT

Various characterization techniques are combined to confirm the successful preparation of UiO‐66‐NH_2_, 6FDA‐TFMB, and UFT. Fourier transform infrared (FT‐IR, Figure 1b) measurements are performed to analyze the chemical structures. The absorption peak at 570 cm^−1^ for UiO‐66‐NH_2_ can be ascribed to the Zr—O coordination bonds formed by ZrCl_4_ and BDC‐NH_2._^[^ ^14^ ^]^ Additionally, the characteristic peaks of —NH_2_ at 3470 and 3360 cm^−1^ also demonstrate the successful synthesis of UiO‐66‐NH_2_. The FT‐IR spectrum of 6FDA‐TFMB exhibits imide characteristic peaks I (1790 cm^−1^), II (1730 cm^−1^), III (1383 cm^−1^) and IV (720 cm^−1^), meanwhile there does not exist any amino stretching vibration, proving the complete imidization. Furthermore, the anhydride characteristic peaks appear at 1860 and 1790 cm^−1^, indicating that 6FDA‐TFMB is anhydride‐terminated. The appearance of Zr—O and imide absorption peaks for UFT manifests that 6FDA‐TFMB has been successfully encapsulated on UiO‐66‐NH_2_. It is noteworthy that the amino characteristic peaks do not disappear completely, owing to the large molecular weight of 6FDA‐TFMB that only reacts with NH_2_ on the surface of UiO‐66‐NH_2_ framework. Therefore, the molecular weight of 6FDA‐TFMB is also investigated by gel permeation chromatography (GPC, Figure 1c and Table S2, Supporting Information), and number average molecular weight (*M*_n_) is about 5.2 × 10^3^ g mol^−1^. Compared with tiny pores of UiO‐66‐NH_2_, such a large molecule could not enter the interior of UiO‐66‐NH_2_, ensuring its high porosity, which is in conformity with the FT‐IR results, that is only the reaction between 6FDA‐TFMB and NH_2_ on the surface of UiO‐66‐NH_2_. Besides, X‐ray diffraction (XRD, Figure 1d) is conducted for analyzing the crystal structure of UiO‐66‐NH_2_ and UFT. The strong and sharp diffraction peaks of UiO‐66‐NH_2_ demonstrate the high crystallinity, and all of them are consistent with previous reports.^[^ ^9^, ^15^ ^]^ Similar sharp peaks with slightly reduced intensity appear in the XRD curve of UFT as compared to that of UiO‐66‐NH_2_, indicating that the amorphous 6FDA‐TFMB encapsulated on the UFT surface decreases the crystallinity. In addition, the surface morphologies of UiO‐66‐NH_2_ (Figure 1e) and UFT (Figure 1f) are observed by scanning electron microscope (SEM). It is found that UiO‐66‐NH_2_ is 50–100 nm of octahedral crystals, and the interface between each nanoparticle is clearly visible. In contrast, UFT are 300–500 nm of clusters consisting of several UiO‐66‐NH_2_ without obvious interface at the junction, indicating that each UiO‐66‐NH_2_ is well fused by 6FDA‐TFMB. Thermogravimetric analysis (TGA) is also employed to assess their thermal stability (Figure 1g), and initial decomposition temperature (*T*_d 5%_) and residual char are illustrated in Table S3, Supporting Information. Both 6FDA‐TFMB and UiO‐66‐NH_2_ exhibit the high thermal stability due to their rigid structures (i.e., aromatic structures and metal Zr). Combining their merits, UFT also shows high *T*_d 5%_ (325.7 °C) and residual char (55.3%). Figure 1h shows the N_2_ adsorption–desorption isotherms of UiO‐66‐NH_2_ and UFT, the BET surface area of UiO‐66‐NH_2_ is 1039 m^2^ g^−1^, which is similar to the results of previous studies,^[^ ^16^ ^]^ while the BET surface area of UFT decreases to 715 m^2^ g^−1^ due to the non‐porous 6FDA‐TFMB on the surface of UFT. The elemental compositions of UiO‐66‐NH_2_ and UFT are tested by XPS (Figure 1i), and the peaks at 183, 285, 400 and 532 eV are attributed to Zr 3d, C 1s, N 1s and O 1s, respectively. Additionally, the F 1s peak at 689 eV demonstrates the successful encapsulation of 6FDA‐TFMB on UiO‐66‐NH_2_. Further analysis of C 1s high‐resolution spectrum of UFT (Figure 1j) reveals that it can be decomposed into C=C (284.5 eV), C—N (285.2 eV), C—C (286.1 eV), and C=O (288.8 eV) as well as a C—F peak at 293.2 eV, which originates from the —CF_3_ group on 6FDA‐TFMB. The above results manifest the successful preparation of UFT.

### 2.2 Optical Properties

**Figure 2:**
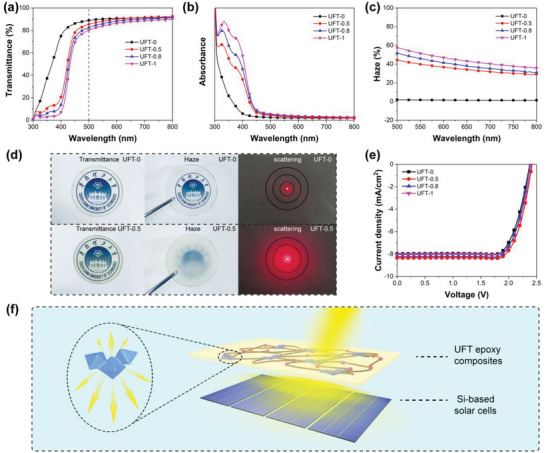
a) Transmittance, b) absorbance, and c) haze of UFT composites. d) Optical photos (in contact with the paper and away from the paper) and light scattering effect of UFT‐0 and UFT‐0.5. e) *J*−*V* curves of solar cells with UFT‐0, UFT‐0.5, UFT‐0.8, and UFT‐1. f) Schematic diagram of the light scattering effect of UFT composites on solar cells.

The optical properties of UFT‐modified epoxy composites are measured by a UV–Vis spectrophotometer, and it is seen that the composites with different UFT contents all maintain high transmittance (>80%) at 500 nm (**Figure** **2**a), which is due to the improved compatibility caused by the reactive 6FDA‐TFMB and the enhanced forward scattering ability enabled by the large size of UFT. Besides, UFT composites exhibit the strong absorption during the UV region as compared to that of UFT‐0 (Figure 2b), and the absorption intensity is gradually increased with the loading of UFT, manifesting the excellent UV blocking ability of UFT composites. The presence of the chromophore in UiO‐66‐NH_2_ enhances the UV absorption, which is not achieved for UiO‐66 without the amino group.^[^ ^17^ ^]^ Haze is also investigated to assess the light scattering of UFT composites (Figure 2c). Compared with the low haze (2%) of UFT‐0, the haze of UFT‐0.5, UFT‐0.8 and UFT‐1 gradually increases to 45%, 52% and, 58% at 500 nm, suggesting that the haze can be tuned by the UFT content. The enhanced haze is mainly due to that 6FDA‐TFMB act as a linker to enlarge the UFT size and a shielding layer to protect UiO‐66‐HN_2_ from penetration by small molecules. The former enhances the forward scattering and the latter maintains the disparity in the optical properties (e.g., refractive index) between the matrix and UFT. Besides, the terminal anhydride groups of 6FDA‐TFMB can covalently bond the epoxy groups and promote the dispersion of UFT in the polymer matrix, which is significant for the preparation of optical materials with uniform haze. Additionally, Figure 2d shows the optical photos of UFT‐0 and UFT‐0.5. Both the images are clearly visible when the composites are in contact with the paper, indicating the high transmittance of UFT composites. When the composites are moved away from the paper, the image underneath UFT‐0.5 becomes turbid as compared to that of UFT‐0, which is due to the enhanced light scattering. Moreover, to observe the change of haze more intuitively, a red laser beam is irradiated through UFT composites on a white paper with circles of different diameters (Figure 2d). A bright spot with a diameter of about 1 cm appears on the paper when the laser passes through UFT‐0, revealing that the light scattering caused by UFT‐0 is negligible. The illuminated area of the paper becomes larger when the laser passes through UFT‐0.5, suggesting a significant enhancement in light scattering ability, which is consistent with the results of haze measurement. To further illustrate the contribution of interface fusion strategy, control materials, including epoxy composites with 0.5 wt% 6FDA‐TFMB (6FT‐0.5) and composites with 0.5 wt% of UiO‐66‐NH_2_ (UiO‐0.5), are prepared and their optical properties are compared with those of UFT‐0.5. 6FDA‐TFMB shows good compatibility with epoxy resin due to its suitable molecular weight and large volume of —CF_3_ side group, thus 6FT‐0.5 possesses high transmittance (Figure S2a, Supporting Information), low haze (Figure S2c, Supporting Information) as well as weak scattering effect (Figure S2e, Supporting Information). UiO‐0.5 shows high transmittance (Figure S2b, Supporting Information) with limited haze (Figure S2d, Supporting Information) compared to those of UFT‐0.5, which is due to the fact that the exposed pore structure of UiO‐66‐NH_2_ is penetrated by small molecules such as the curing agent MHHPA, weakening the light scattering ability. In contrast, the surface of UFT‐0.5 is protected by 6FDA‐TFMB, retaining the internal porosity and the high scattering ability. Consequently, the forward scattering effect of UFT‐0.5 is also stronger than that of UiO‐0.5 (Figure S2f, Supporting Information).

Owing to the high transparency, tunable haze and excellent anti‐ultraviolet ability, UFT composites are explored for the applications in silicon solar devices. Reflection loss is one of the major losses of silicon‐based solar cells. Thus, some techniques, such as texturing, have been applied to improve the light capture capability of crystalline silicon. Typically, the honeycomb‐like structure produced by acid in multicrystalline silicon (mc‐Si) cells is less efficient in the light capture capability than the pyramidal‐shaped light trapping structure generated by alkali in monocrystalline silicon cells, resulting in inferior PCEs. Therefore, the light trapping capability of mc‐Si cells needs to be further improved. Previous reports have revealed that various nanostructures are promising in improving the light trapping by increasing the light path, but most of which are not based on silicon cells. Here, UFT composites are applied to mc‐Si cells as encapsulation materials (Figure 2f) with the following functions: i) as a refractive matching layer between air and silicon solar cells, and UFT composites can capture the broadband light at a wide angle; and ii) the high transmission and forward scattering effect of the MOF clusters increases the incident angle of the light on the surface of silicon, while decreases the reflection, promoting multiple diffusion of light in the solar cells and increasing the light path. This leads to an improvement in the number of photons captured by the active layer, especially for the long wavelengths which possess smaller absorption coefficients. In the case of solar cell *J*–*V* measurements, this strategy has the same effect as increasing the illumination light intensity. Theoretically, light intensity increase will lead to the direct increase in *J*_sc_ (*J*_sc_ generally increase linearly with light intensity) and the slight increase in *V*_oc_ (following an exponential function), which eventually leads to an increase in PCE. To verify this conjecture, UFT composites with various UFT loadings are adhered to the silicon‐based solar cells and their PCE are investigated (Figure 2e and Table S4, Supporting Information), the PCE of solar cells with UFT‐0.5, UFT‐0.8 and UFT‐1 are all increased, and the better one is UFT‐0.5 with a PCE of 15.64%, which shows a 6.6% enhancement as compared to that of UFT‐0 (14.67%). *J*_sc_ also increases from 7.98 to 8.35 mA cm^−2^. However, higher UFT content could not further improve PCE probably due to the decreased light transmission. Furthermore, the excellent anti‐UV ability of UFT composites can filter out the UV rays of the sun, which is conducive to extending the lifetime of solar cells. The above results manifest the promising applications of UFT composites in the light management of solar cells.

### 2.3 Dielectric and Hydrophobic Properties

**Figure 3:**
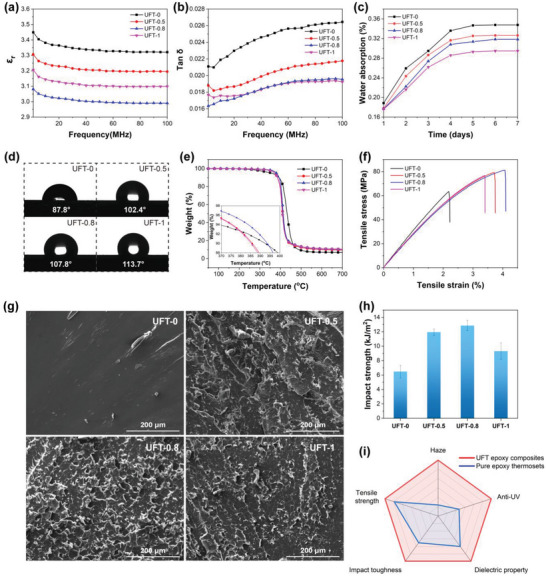
a) Dielectric constant (*ε*_r_) and b) dielectric loss tangent (tan*δ*) of UFT composites at 1–100 MHz. c) Water absorption and d) water contact angles of UFT composites. e) TGA plots of UFT composites under N_2_ atmosphere. f) Tensile properties of UFT composites. g) SEM micrographs of the fracture surfaces and h) impact properties of UFT composites. i) A radar plot showing a comparison between UFT epoxy composites and pure epoxy thermosets.

Dielectric property is another important parameter of encapsulation materials, and the dielectric constant (*ε*_r_) and dielectric loss tangent (tan*δ*) of the UFT epoxy composites are measured from 1 to 100 MHz. It is found that the *ε*_r_ (**Figure** **3**a) and tan*δ* (Figure 3b) of the epoxy composites with various UFT loadings are lower than those of UFT‐0 during the tested ranges. When the UFT content is 0.8 wt%, the *ε*_r_ and tan*δ* are decreased to 3.08 and 0.016 (at 1 MHz), respectively, as compared to those (3.45 and 0.021) of UFT‐0. In addition, the dielectric properties of UFT‐0 and UFT‐0.8 are also tested in the range of 9–12 GHz. As revealed in Figure S3, Supporting Information, the tan*δ* of UFT‐0.8 at 10 GHz is decreased from 0.023 (UFT‐0) to 0.018. These results are attributed to the unique core–shell structure of UFT through interface fusion strategy. Not only does UFT combine the high porosity of UiO‐66‐NH_2_ (*ε*_r_ = 2.0 at 1 MHz)^[^ ^6c^ ^]^ and the low dielectric fluorine‐containing imide oligomer 6FDA‐TFMB (*ε*_r_ = 2.8 at 1 MHz),^[^ ^18^ ^]^ but also 6FDA‐TFMB on the surface of UFT can prevent small molecules from the penetration into UFT, ensuring the high porosity of UiO‐66‐NH_2_. In addition, according to the epoxy curing mechanism, the reaction of amino groups and epoxy groups will generate high polar hydroxyl groups, which is detrimental to the improvement of dielectric properties, and an effective way is to replace amino curing agents with anhydride (see Figure S4, Supporting Information). After reacting with UiO‐66‐NH_2_, anhydride‐terminated 6FDA‐TFMB converts some amino groups of UiO‐66‐NH_2_ into low‐polarity imide moieties, and the anhydride groups on the UFT surface can further react with the epoxy groups and chemically bond with the epoxy matrix, promoting dispersion and reducing interfacial polarization as well as avoiding the generation of high polar hydroxyl groups. Moreover, the rigid structure of UiO‐66‐NH_2_ and the bulky —CF_3_ group on the 6FDA‐TFMB increase the steric hindrance effect, which hinders the movement of epoxy segments and also reduces the excessive cross‐link density of epoxy thermosets, thus increasing the free volume and contributing to the improved dielectric properties. However, the higher UFT loading cannot further decrease the dielectric constant, suggesting that the higher interfacial polarization and the polarity of the UiO‐66‐NH_2_ structure play a dominant role, thus, composites with higher UFT contents are expected to possess higher dielectric constants according to the additive principle. The decrease in dielectric loss tangent is attributed to the fact that Zr^4+^ in UiO‐66‐NH_2_ does not easily cause electronic effects^[^ ^12a^ ^]^ as well as the low dielectric loss (<0.01 at 1 MHz) of 6FDA‐TFMB.^[^ ^18^ ^]^ As a strong polar molecule, water possesses a very high *ε*_r_ (about 78) and influences enormously the dielectric properties of the materials, thus it is very crucial to assess the hydrophobic properties of UFT‐modified epoxy thermosets. Figures 3c,d illustrate the water absorption and water contact angles of UFT composites, respectively. The hydrophobic properties of the UFT composites are progressively improved with increasing the UFT contents. UFT‐1 displays a low water absorption of 0.29% and a high contact angle of 113.7° as compared to those (0.35% and 87.8°) of UFT‐0, which may be owing to the unique core–shell structure of UFT preventing the penetration of water, and the low surface energy and low‐polarity imide structures of 6FDA‐TFMB containing fluorine groups (—CF_3_).

### 2.4 Thermal and Mechanical Properties

Encapsulation materials also require excellent thermal and mechanical properties to provide protection for the devices. Therefore, the thermal properties of the epoxy thermosets with different UFT contents are tested by TGA (Figure 3e and Table S5, Supporting Information). UFT‐0.5, UFT‐0.8, and UFT‐1 exhibit higher *T*_d 5%_ and residual char (*R* at 700 °C) than UFT‐0. When the UFT content is 0.8 wt%, the *T*_d 5%_ and *R* of UFT‐0.8 reached 380.0 °C and 10.3%, which is ascribed to the high thermal stability and residual char of UFT. While higher UFT content (1 wt%) may be detrimental to *T*_d 5%_, probably due to the agglomeration of UFT particles and the decrease in crosslink density of the epoxy thermosets, which is consistent with the results of the previous works.^[^ ^12a^ ^]^ In addition, the mechanical properties of the UFT composites are also investigated (Figure 3f,h and Table S6, Supporting Information). The tensile and impact properties of the UFT‐modified epoxy composites are all enhanced and UFT‐0.8 possesses the excellent overall performance (81.2 MPa of tensile strength and 12.9 kJ m^−2^ of impact toughness), mainly owing to: i) the rigid structure of UiO‐66‐NH_2_ not only facilitates the enhancement of tensile strength, but also provides blocking and dispersing effect on crack expansion during the fracture of the thermosets, which is known as “crack pinning”, and thus enhances the toughness;^[^ ^19^ ^]^ ii) the anhydride‐terminated 6FDA‐TFMB on the surface of UFT is chemically bonded with the epoxy groups to increase the compatibility of UFT and epoxy matrix, which is also conducive to the enhancement of mechanical properties; and iii) UFT optimizes the crosslink density of the epoxy thermosets to increase the toughness. However, higher UFT loading induces an agglomeration effect and thus deteriorates the mechanical properties, which is analogous to the thermal properties. Moreover, the fracture morphology of UFT‐modified epoxy thermosets after impact measurement is observed by scanning electron microscope (SEM) (Figure 3g). The fracture surface of UFT‐0 is flat and smooth with only a few tiny cracks, indicating the typical brittle feature. In contrast, the fracture surface morphology becomes rough and uneven after UFT modification, and more crimped and mosaic flakes appear on the fracture surface of UFT‐0.8 as compared to that of UFT‐0.5 or UFT‐1, manifesting that the appropriate UFT content can significantly enhance the toughness of the epoxy thermosets, which is in conformity with the results of mechanical measurement. Finally, Figure 3i provides a comprehensive comparison of the haze, anti‐UV, dielectric property (1/tan*δ* at 10 GHz), impact toughness, and tensile strength of UFT epoxy composites and pure epoxy thermosets. UFT epoxy composites show excellent overall performance (i.e., optical, dielectric, and mechanical properties) compared to that of pure epoxy thermosets. To further demonstrate the performance advantages of UFT epoxy composites, Table S7, Supporting Information, compares the comprehensive properties of UFT‐0.8 with those of commonly used commercial solar cell encapsulation materials (glass and EP‐AB). Compared with glass and EP‐AB, UFT‐0.8 simultaneously exhibits a high transmittance, a higher haze, and an excellent UV resistance. The dielectric properties and tensile strength of UFT‐0.8 are also superior to those of the other two encapsulation materials. UFT‐0.8 also possesses a lower water absorption compared to EP‐AB. The above comparisons suggest that UFT composites can satisfy the requirement of high‐performance encapsulation materials for energy systems and microelectronics industries.

## 3 Conclusions

In this paper, we design a novel MOF cluster with enhanced forward scattering and robust porosity through interface fusion strategy, and employ it to prepare UFT epoxy composites with high transmittance (>80%), tunable haze (45–58%) and improved dielectric properties. UFT epoxy composites show efficient light management, resulting in a significant enhancement in PCE of silicon solar cells. In addition, UFT epoxy composites also possess excellent anti‐UV, hydrophobic, thermal, and mechanical properties. Therefore, UFT composites are promising for encapsulation materials in energy systems and microelectronics industries. The concept of assembling nanoparticles into cluster structures through engineering and fusing their interfaces provides a novel and facile approach for the synthesis of covalent bond‐mediated multifunctional nanostructures as well as the simultaneous achievement of efficient optical management and excellent dielectric properties in encapsulation systems.

## 4 Experimental Section

### Materials

Zirconium chloride (ZrCl_4_, 98%) and 2,5‐dicarboxyaniline (BDC‐NH_2_, 98%) were obtained from Aladdin Reagent Co., Ltd., China. 4,4″‐(hexafluoroisopropylidene)diphthalic anhydride (6FDA, 98%), 2,2″‐bis(trifluoromethyl)benzidine (TFMB, 98%), methylhexahydrophthalic anhydride (MHHPA, 99%), and 2,4,6‐tris(dimethylaminomethyl)phenol (DMP‐30, 95%) were purchased from Beijing InnoChem Science & Technology Co., Ltd. Diglycidyl ether of bisphenol A (DGEBA, T.P.) with the epoxy value of 0.51 was supported by Baling Petrochemical Co., Ltd., China. 6101 (A/B) Epoxy resin (EP‐AB, T.P.) was provided by TONSIN LIDA Co., Ltd., China. Tempered glass (thickness of 1 mm) was supported by Merck Co., Ltd., USA. *N*,*N*‐dimethylformamide (DMF, 99.5%), acetic acid (HAc, 99.5%), benzoic acid (99.5%), toluene (99.5%), *n*‐hexane (97%), methanol (99.5%), and dichloromethane (DCM, 99.5%) were supplied by Richjoint Co., Ltd., China.

### Preparation of UiO‐66‐NH_2_

ZrCl_4_ (0.350 g), BDC‐NH_2_ (0.272 g), DMF (140 mL) and HAc (5.15 mL) were added to the hydrothermal kettle, then sonicated until the solution was clarified and placed in an oven at 120 °C for 24 h. The precipitate was washed 3 times with DMF to remove excess reactants and centrifuged at 10 000 rpm for 10 min, followed by being washed with methanol to remove residual DMF. UiO‐66‐NH_2_ was obtained after being dried in vacuum at 100 °C for 24 h.

### Preparation of Anhydride‐Terminated 6FDA‐TFMB

6FDA (5.331 g), benzoic acid (17.5 g), and HAc (50.0 mL) were added to a three‐neck flask and stirred at 120 °C for 1 h under nitrogen. Then TFMB (2.562 g) was added and the mixture was stirred at 120 °C for 5 h. After the reaction was completed, the product was cooled to room temperature and precipitated using *n*‐hexane. The precipitate was filtered, washed twice with *n*‐hexane, dried in vacuum at 120 °C for 1 day, and 6FDA‐TFMB was obtained.

### Preparation of UFT

UiO‐66‐NH_2_ (0.5 g), anhydride‐terminated 6FDA‐TFMB (6.5 g) and DMF (60 mL) were added to a three‐neck flask and sonicated until UiO‐66‐NH_2_ was well dispersed, followed by a reaction at room temperature for 72 h under nitrogen, then toluene (10 mL) was added and the suspension was refluxed at 120 °C for 24 h. After cooling to room temperature, the suspension was centrifuged, and the precipitate was washed twice with DCM to remove the unreacted 6FDA‐TFMB, dried under vacuum at 120 °C for 24 h, and UFT was obtained.

### Preparation of Solar Cells with UFT‐Modified Epoxy Thermosets

The formulation of UFT‐modified epoxy thermosets is displayed in Table S1, Supporting Information, taking the preparation of epoxy composite with 0.8 wt% UFT content as an example. UFT (0.075 g) and epoxy prepolymer DGEBA (5.0 g) were uniformly dispersed in 20 mL of DCM, then heated to 60 °C to remove most of the solvent. After that, the curing agent MHHPA (4.285 g) and accelerator DMP‐30 (0.047 g) were added under stirring. After air bubbles were removed at 60 °C in vacuum, the mixture was poured into a mold and the epoxy prepolymer was cured according to the curing procedure (120 °C for 2 h, 140 °C for 4 h, 160 °C for 2 h) to obtain the epoxy thermoset UFT‐0.8. Other epoxy thermosets with different UFT contents were prepared by similar processes. Synthetic schemes of 6FDA‐TFMB, UFT, and UFT epoxy composites are shown in Figure S1, Supporting Information. The surfaces of the bare multicrystalline silicon were washed several times with deionized water, alcohol, and acetone. Subsequently, four mc‐Si solar cells with a total active area of 5 cm^2^ were merged on a printed circuit board (PCB) and encapsulated with UFT composites. The schematic diagram of preparation process of UFT composites is illustrated in Figure 1a, and the detailed characterizations are given in Supporting Information.

## Conflict of Interest

The authors declare no conflict of interest.

## Author Contributions

M.H.W. and W.Q.X conceived the ideas and designed the experiments. M.H.W, J.X.L., and X.Z. performed the experiments. All authors discussed the results and contributed to writing the paper.

## Supporting information

Supporting Information

## Data Availability

The data that support the findings of this study are available from the corresponding author upon reasonable request.
