# Supplementary material for: Design of Imide Oligomer‐Mediated MOF Clusters for Solar Cell Encapsulation Systems by Interface Fusion Strategy
Source: Adv Sci (Weinh). 2023 Jan 29;10(9):2206748. doi: 10.1002/advs.202206748 (PMC10037975; doi:10.1002/advs.202206748)
Supplement: Supplementary file 1 — Supporting Information [file ADVS-10-2206748-s001.pdf]

Supporting Information

Design of Imide Oligomer-Mediated MOF Clusters for Solar Cell Encapsulation Systems by Interface Fusion Strategy

Minghong Wu[a], Jiaxin Li[a], Xi Zhang[a], Shumei Liu[a], Jianqing Zhao*[a], and Weiqi Xie*[b]

[a]School of Materials Science and Engineering, State Key Laboratory of Luminescent Materials and Devices, South China University of Technology, Guangzhou, 510640, P. R. China

[b]School of Minerals Processing and Bioengineering, Central South University, Changsha, 410083, P. R. China

Corresponding authors

*Tel./Fax: +86-15913171507. E-mail: weiqixie@csu.edu.cn (Weiqi Xie)

*Tel./Fax: +86-13609724000. E-mail: psjqzhao@scut.edu.cn (Jianqing Zhao)

Characterizations

Fourier transform infrared (FT-IR) spectra were achieved on a Vertex70 spectrometer (Bruker) from 400 cm^{-1} to 4000 cm^{-1}. The molecular weight and distribution of 6FDA-TFMB were investigated by gel permeation chromatography (GPC, Waters) with a THF eluent. X-ray diffraction (XRD) was measured by a X-ray diffractometer (Bruker D8) from 5° to 60° (2θ). The morphology was observed by scanning electron microscope (SEM, Zeiss Merlin). Thermogravimetric analysis (TGA) was conducted by TG-209F1 TGA (Netzsch) from 30 °C to 800 °C under nitrogen atmosphere. N_2 adsorption-desorption isotherms were tested by a fully automated specific surface area and porosity analyzer (Micromeritics ASAP 2460). X-ray photoelectron spectroscopy (XPS, Thermo Scientific K-Alpha) was performed to investigate the elemental compositions. The optical properties were collected by a UV-Vis spectrophotometer (HITACHI U-3900H). *J-V* curves were measured by a Keithley 2400 sourcemeter (TEKTRONIX) under one sun illumination (AM 1.5G, 100mW cm^{-2}) with a scan speed of 100 mV s^{-1}. Dielectric properties were assessed by a HP4284A capacitance analyzer (Agilent) from 1 MHz to 100 MHz, and those at high frequency were investigated by Microwave vector network analyzer PNA-N5244A (Agilent) from 9 to 12 GHz with waveguide method. Water absorption was evaluated by placing samples to deionized water at room temperature for 7 days according to ASTM D570-98 (2018) standard. Water contact angle was recorded by DSA100 (KRUSS) at room temperature. Tensile properties were analyzed using Instron-5967 testing machine according to ASTM D638-08 standard. Impact properties were investigated using Zwick-5113 impact testing machine according to ASTM D256-10 standard.

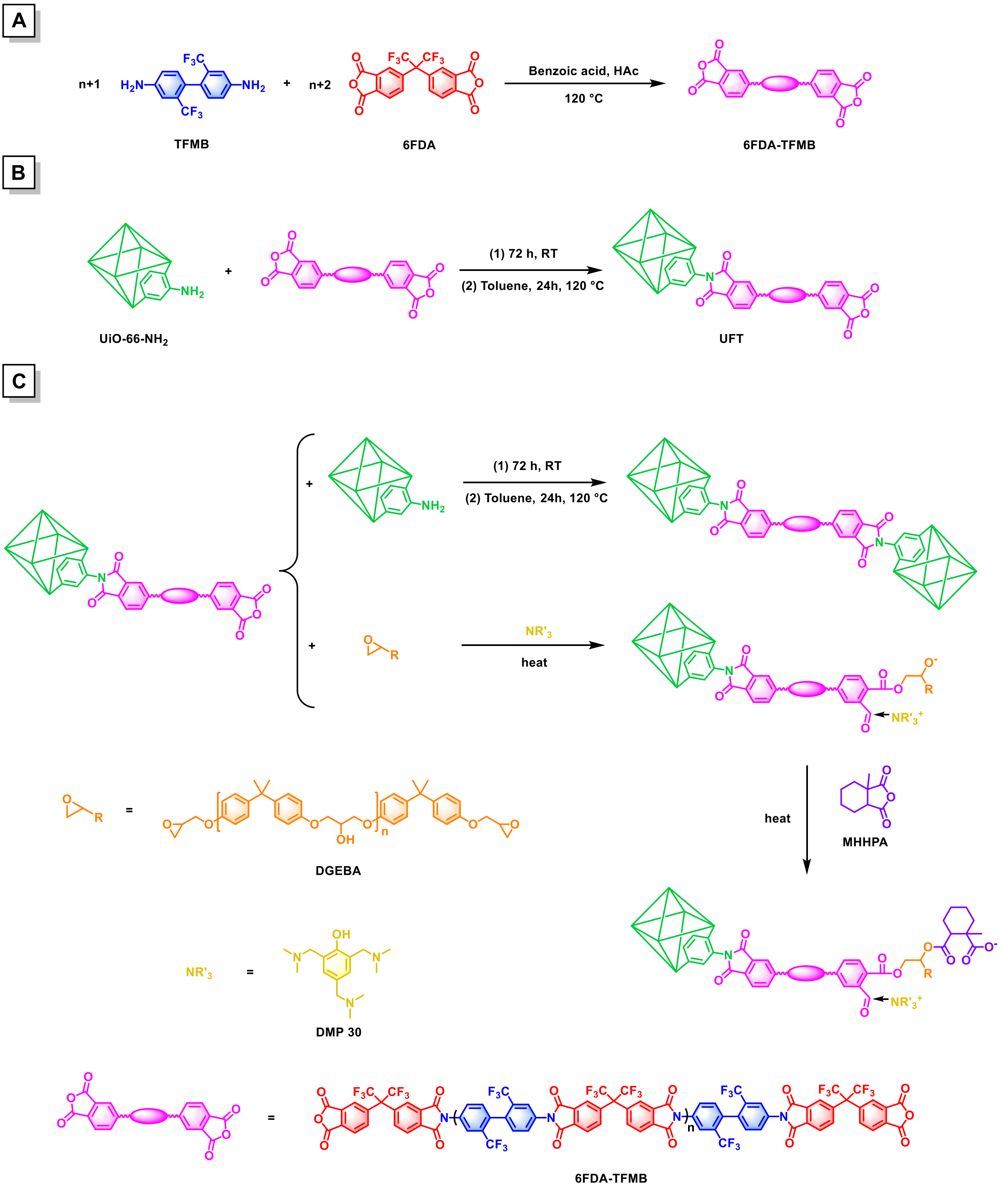

Fig. S1 Synthetic routes of 6FDA-TFMB, UFT, and UFT epoxy composites

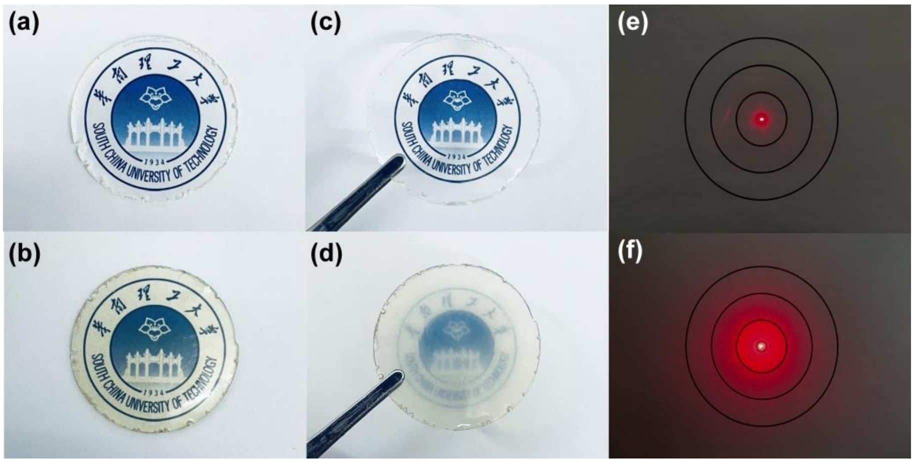

Fig. S2 Optical images of (a) 6FT-0.5 and (b) UiO-0.5 in contact with the paper. Optical images of (c) 6FT-0.5 and (d) UiO-0.5 away from the paper. Scattering effects of (e) 6FT-0.5 and (f) UiO-0.5

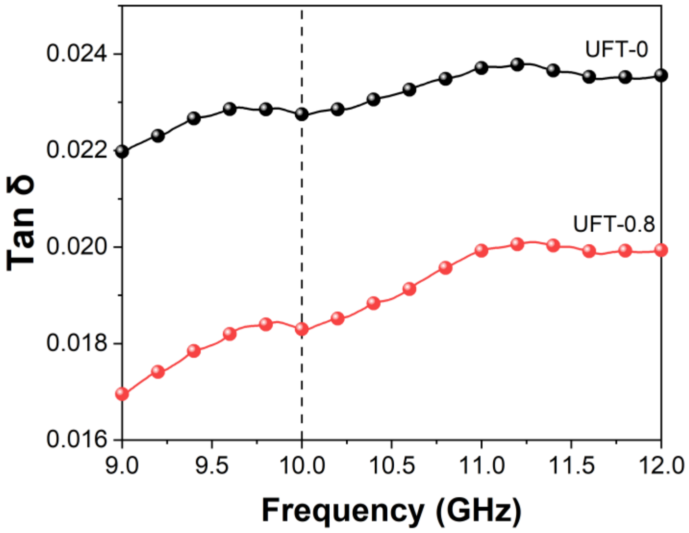

Fig. S3 Tan δ of UFT-0 and UFT-0.8 at 9 GHz - 12 GHz

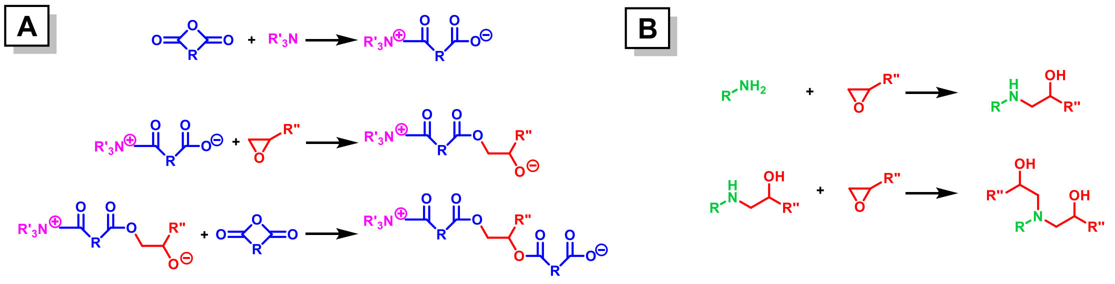

Fig. S4 Comparison of the reaction mechanism of (A) anhydride and (B) amino groups with epoxy groups

Table S1 Stoichiometric formulation of UFT-modified epoxy system

Samples*	DGEBA (wt%)	MHHPA (wt%)	UFT (wt%)	DMP-30 (wt%)
UFT-0	53.59	45.91	0	0.5
UFT-0.5	53.32	45.68	0.5	0.5
UFT-0.8	53.16	45.54	0.8	0.5
UFT-1	53.05	45.45	1	0.5

* Sample name: UFT-X, X represents the content (wt%) of UFT.

Table S2 GPC results of imide oligomers 6FDA-TFMB

	M_n ($\times 10^3$g/mol)	PDI
1	9.3	1.13
2	5.2	1.01
3	3.6	1.01
4	2.1	1.03

Table S3 Thermal properties of UiO-66-NH_2, 6FDA-TFMB and UFT

Samples	$T_{d\ 5\%}$ (°C)	Residual char at 800 ℃ (%)
UiO-66-NH_2	383.0	47.1
6FDA-TFMB	283.8	61.0
UFT	325.7	55.3

Table S4 Some parameters of solar cells with UFT-0, UFT-0.5, UFT-0.8, and UFT-1

Samples	PCE (%)	FF (%)	J_{sc} (mA/cm^2)	V_{oc} (V)
UFT-0	14.67	77.31	7.98	2.38
UFT-0.5	15.64	77.50	8.35	2.42
UFT-0.8	15.23	77.74	8.23	2.38
UFT-1	15.03	77.83	8.04	2.40

Table S5 Thermal properties of UFT composites

Samples	$T_{d\ 5\%}$ (°C)	Residual char at 700 ℃ (%)
UFT-0	355.7	6.8
UFT-0.5	371.6	9.5
UFT-0.8	380.0	10.3
UFT-1	371.1	10.5

Table S6 Mechanical properties of UFT composites

Samples	Tensile strength (MPa)	Elongation at breaking (%)	Impact strength (kJ/m^2)
UFT-0	63.4 ± 1.12	2.19 ± 0.16	6.48±0.89
UFT-0.5	79.2 ± 1.40	3.72± 0.13	11.95±0.45
UFT-0.8	81.2 ± 1.83	4.08 ± 0.19	12.86±0.72
UFT-1	76.8 ± 1.44	3.39± 0.11	9.31±1.14

Table S7 Comparison of the properties of UFT-0.8, EP-AB and glass

Samples	Transmittance at 500 nm	Haze at 500 nm	Anti-UV Abs at 350 nm	ε_r at 1MHz	Tensile (MPa)	Water absorption (%)
UFT-0.8	83%	52%	1.33	3.1	81	0.32
EP-AB	92%	2%	0.07	4.6	20	1.18
Glass	90%	1%	0.05	5.8	42	0.01
